# Supplementary material for: Vascular dysfunction is associated with major adverse cardiovascular events in prediabetes: A cohort study
Source: PLoS One. 2025 Jun 5;20(6):e0324945. doi: 10.1371/journal.pone.0324945 (PMC12140275; doi:10.1371/journal.pone.0324945)
Supplement: Attachment — Submitted filename: Response to Reviewers.docx [file pone.0324945.s002.docx]

**Response to reviewers**

We want to thank the editors and reviewers for their thoughtful review of our manuscript entitled: **Vascular dysfunction is associated with major adverse cardiovascular events in prediabetes.** Below is a point-by-point response to the critiques.

**Additional Editor Comments**

Jalal Diana et al. performed a subanalysis of the Framingham Offspring Study and the Generation III cohort to evaluate differences in vascular dysfunction parameters (brachial artery flow-mediated dilation (BA-FMD), aortic stiffness via carotid-femoral pulse wave velocity (cfPWV), and coronary artery calcium (CAC)), predictors of these conditions, and whether vascular dysfunction predicts incident MACE events. The authors found that patients with prediabetes exhibited impaired vascular hemodynamics, tested several predictors of these conditions, and concluded that BA-FMD and CAC predict MACE events. This is a well-structured study with potential implications for diabetes care. However, together with three external referees, we identified several concerns that should be addressed before a final decision on this manuscript is made. Below are my suggestions:

1. **In the abstract, please include the mean age and the proportions of women and men as part of the descriptive characteristics of the studied sample.**

**Response**

We have added the mean age and proportions of men and women to the descriptive characteristics in the abstract.

**Amendment**

Methods section of abstract:

“We conducted an observational study of 5742 adults (age 54.9 ± 11.5 years, 54% female) in the Framingham Offspring and Generation III cohorts.”

2. **The objective is stated in a very broad and untargeted way. I recommend the authors restructure the study aims using the PECOT format to specifically mention the primary and secondary objectives.**

**Response**

We have reformatted our study aims using PECOT formatting.

**Amendment**

Introduction paragraph 2:

“This study aimed to assess the relation between BA-FMD, cfPWV, and CAC score, as measures of vascular dysfunction, with future MACE in adults with prediabetes compared to normoglycemic adults.”

3. **Please include the STROBE checklist for observational studies as a supplementary appendix.**

**Response**

The STROBE checklist for observational studies has been added as a supplementary appendix.

**Amendment**

See the supplementary appendix.

4. **I suggest rephrasing “history of CKD” to “decreased eGFR” to ensure precision in describing this metric.**

**Response**

We have rephrased “history of CKD” to “CKD defined by eGFR” for improved precision in wording. Changes were made in the methods (clinical characteristics and outcome measurements), methods (statistical analysis), and results (participant characteristics) sections as well as in Table 1.

**Amendment**

Materials and methods clinical characteristics and outcomes measures:

“Participant age, sex, race, body mass index (BMI), history of CVD, estimated glomerular filtration rate (eGFR), and smoking status were obtained from baseline visits within the Framingham Offspring Study and Framingham Generation 3 cohorts.”

“eGFR was calculated via the 2021 CKD-EPI creatinine equation, and chronic kidney disease (CKD) was defined as eGFR <60 at baseline visit (23).”

5. **Please justify the rationale for grouping the CAC scores into 0, 1–100, and ≥100. Were patients with CAC scores between 0 and 1 included in the study?**

**Response**

Thank you for bringing this to our attention. Participants with CAC scores between 0 - 1 were included in the study. More accurately defined, the CAC grouping utilized in our study was 0, >0-100, >100. We have changed our manuscript to accurately reflect this. Changes were made in the following sections: methods (clinical characteristics and outcome measurements), results (vascular function and CAC score in prediabetes), results (MACE in prediabetes), Table 3, and Figure 1.

**Amendment**

Materials and methods clinical characteristics and outcomes measures:

“CAC score was then grouped into the following categories: 0, >0-100, >100.”

Results vascular function and CAC score in prediabetes:

“As shown in Fig 1C, 60% of participants in the normoglycemia group had a CAC score of 0, 22% had a CAC score of >0-100, and 18% had a CAC score >100, while in the prediabetes group, 44% had a CAC score of 0, 23% had a CAC score of >0-100, and 33% had a CAC score >100.”

Results MACE in prediabetes

“Additionally, higher cfPWV was associated with a 16% higher risk of MACE. Furthermore, a CAC score of >0-100 was associated with a 173% greater risk of MACE, and a CAC score of >100 was associated with a 11.3 times higher risk of MACE (HR [95% CI], 11.3 [6.71,19.0]; p<0.001).”

Table 3

We have adjusted CAC score labeling to read “>0-100” rather than 1-100.

Figure 1:

We have adjusted the legend of panel c to read “>0-100” rather than 1-100.

6. **Include a reference to justify the choice of incident MACE events as an outcome. Most MACE definitions exclude “all-cause mortality” and instead focus on “CVD-related mortality.”**

**Response**

We recognize that many studies use a definition of MACE that focuses on CVD-related mortality. As recognized by a 2021 systematic review by Bosco et al. (BMC Medical Research Methodology. 2021 Nov 6;21(1):241), there are several definitions of MACE utilized by peer-reviewed articles. For example, they note that MACE is defined as acute myocardial infarction, stroke, or cardiovascular death in about 8.6% of the studies they reviewed, while 13.8% of the studies they reviewed used acute myocardial infarction, stroke, and all cause death as their definition of MACE. We have added this reference in the manuscript.

**Amendment**

Materials and methods clinical characteristics and outcomes measures:

“MACE was defined as incident CHD, stroke, and all-cause mortality after the defined baseline visit (27).”

7. **Did the authors encounter missing data? This is not specified in the statistical plan or the methods section.**

**Response**

Yes, missing data was encountered during our study. Participants were not excluded from the study on the account off missing variables unless they were missing both baseline A1c and fasting glucose values, and were therefore not able to be grouped into normoglycemia or prediabetes categories. We have clarified this in the methods section. Additionally, we have adjusted Table 1 to provide the number of missing values for each of the clinical characteristics. For multivariate models, observations that are missing were not included in the model.

**Amendment**

Materials and methods clinical characteristics and outcomes measures:

“Participants missing baseline clinical characteristics were not excluded from analysis unless both baseline fasting glucose and HbA1c values were missing”.

Results:

Please note that the clinical characteristics in Table 1 have been adjusted to reflect the number of missing values for each characteristic.

8. **Although the AIC method is a valid statistical technique to identify predictors, it is often untargeted and may not yield the best physiopathological insights. I strongly recommend that the authors adopt a more structured approach, such as using directed acyclic graphs (DAGs), to test and support their predictors.**

**Response**

We recognize that AIC is an untargeted statistical approach, especially as we did not fix any specific variables of interest in our models. In considering this critique, we were reminded by our biostatistical team that the intention of our analysis was to identify variables that may contribute to vascular function in prediabetes rather than to identify a direct pathophysiological pathway using variables from clinical practice. Based on this feedback, we have opted to maintain our current analytical approach. We hope that this is acceptable to the editors and reviewers.

9. **Please include the R² values for the models used to evaluate predictors of vascular dysfunction.**

**Response**

We have added R² values for the stepwise selection models used to evaluate predictors of BA-FMD and cfPWV. We were unable to generate R^2^ values for the CAC model as our outcome data was ordinal rather than continuous. These are reported in Table 2.

**Amendment**

See Table 2.

10. **Regarding the Cox regression models, did the authors test the proportional hazards assumption? This can be evaluated using Schoenfeld residuals.**

**Response**

We did not initially test the proportional hazards assumption. We agree that this would add to the completeness of our analysis. As suggested, we have evaluated the proportional hazards assumption using Schoenfeld residuals and updated the study accordingly.

**Amendment**

Materials and methods statistical analysis:

“Hazard ratios and their corresponding confidence intervals and p-values were provided. The proportional hazards assumption was evaluated using Schoenfeld residuals. Due to the robust sample size of our study, a more stringent cutoff of p = 0.01 was used in this context, with Schoenfeld residual p-values greater than 0.01 signifying that Cox proportional hazards modeling was an acceptable framework.”

Results Mace in prediabetes:

“All time-to-event models, both adjusted and unadjusted, had Schoenfeld residual p-values greater than 0.01.”

11. **Consider other potential confounders in the studied relationships, such as BMI, eGFR, and A1C. These could be included in an additional model, even as part of a sensitivity analysis.**

**Response**

This is an important point. We assume that this critique pertains to the association between measures of vascular dysfunction and MACE (Table 3- Cox regression models), as the stepwise selection models in Table 2 included the following variables: age, sex, history of cardiovascular disease, CKD, smoking status, diastolic blood pressure, systolic blood pressure, BMI, A1c, creatinine, fasting glucose, total cholesterol, triglycerides, LDL, HDL, and eGFR. In the Cox regression models, the following variables were included in the adjusted model originally: age, sex, smoking status, systolic blood pressure, BMI, and LDL. In the revised manuscript, we have additionally adjusted for eGFR and included this in the revised Table 3. Of note, after the addition of eGFR to the model, cfPWV no longer predicted MACE in prediabetes. This has been updated in the abstract and manuscript. We also considered sensitivity analysis for CKD, however, considering the small number of participants with CKD, we felt this would not be informative.

In addition to adjusting for eGFR, we added fasting glucose in an additional separate model. We found that this did not modify our findings, and have stated that in the results section.

Finally, we explored the potential effects of age, sex, and prediabetes definition by evaluating interaction terms for the relation between age, sex, or prediabetes definition (fasting glucose or HbA1c) in a separate set of models that evaluated the association between measures of vascular dysfunction and MACE. The inclusion of these interaction terms did not alter the relation between measures of vascular dysfunction and MACE. BA-FMD and CAC >100 continued to significantly predict the MACE in prediabetes. Of note only one of the tested interaction terms was significant in the models – the age and cfPWV interaction term was significant (p=0.02), suggesting that the cfPWV and MACE association is weaker for older individuals. All other interaction terms were ≥ 0.05.

**Amendment**

See Table 3.

Abstract Results:

“After adjusting for traditional cardiovascular risk factors, BA-FMD (HR [95% CI], 0.93 [0.90,0.97]; p<0.001) and CAC score >100 [HR [95% CI], 4.15 [2.24, 7.70]; p<0.001)] were associated with MACE in prediabetes while cfPWV was not (p=0.051).”

Abstract Conclusion:

“Vascular dysfunction measured by BA-FMD independently associates with MACE in prediabetes.”

Materials and methods statistical analysis:

“Interaction terms for age, sex, or prediabetes definition and vascular function or CAC were evaluated in an additional set of models.”

Results section MACE in prediabetes:

“After adjustment for age, sex, smoking status, systolic blood pressure, BMI, and LDL, BA-FMD and CAC score > 100 remained significantly associated with MACE (Table 3). Additional adjustment for fasting glucose or the inclusion of interaction terms for age, sex, or prediabetes definition did not attenuate this relation.”

Discussion Paragraph 1:

“Additionally, endothelial dysfunction and CAC score > 100 were associated with future MACE in prediabetes after adjusting for confounding variables.”

Discussion Paragraph 3:

“Our study expands upon these findings by demonstrating that aortic stiffness was not associated with MACE in prediabetes after adjusting for traditional cardiovascular risk factors in our cohort.”

12. **Please include the median follow-up time and the interquartile range (IQR). Furthermore, estimate the incidence rate of MACE events in the studied population.**

**Response**

We have added median follow-up time (and interquartile range) as well as the incidence rate of MACE events to the results section. We have also included median time-to-event.

**Amendment**

Results Participant Characteristics:

“Median follow up time was 13.2 years (IQR 12.2 – 14.1).”

Results Mace in prediabetes:

“The incidence rate of MACE events in the overall population was 21%. The median time-to-event/loss to follow-up for participants was 12.7 years for the prediabetes group, 13.4 years for the normoglycemia group and 13.1 years for the two groups combined.”

13. **It would be valuable if the authors tested whether different prediabetes definition criteria (e.g., fasting glucose vs. normoglycemia, HbA1c only vs. normoglycemia, and both vs. normoglycemia) individually predict MACE outcomes. This information would be particularly useful for clinicians, especially in low-resource settings where HbA1c is not always available.**

**Response**

We appreciate this suggestion and have considered it. However, in evaluating the literature, we found several manuscripts that had evaluated the risk of CVD based on different definitions of prediabetes (1,2). Importantly, a prior study by Echouffo-Tcheugui et al 2021 (3), evaluated cardiovascular risk by prediabetes definition. They found that prediabetes defined by HbA1c is more specific and captures a higher cardiovascular risk population while fasting glucose is more sensitive and captures a lower cardiovascular risk population. Considering that these findings have been previously noted in the literature, we opted not to incorporate this analysis into the manuscript. We hope that the reviewer/editors find this acceptable.

**References**

(1) Cai X, Zhang Y, Li M, Wu JH, Mai L, Li J, et al. Association between prediabetes and risk of all cause mortality and cardiovascular disease: updated meta-analysis. BMJ. 2020 Jul 15;370:m2297.

(2) Ford ES, Zhao G, Li C. Pre-Diabetes and the Risk for Cardiovascular Disease: A Systematic Review of the Evidence. Journal of the American College of Cardiology. 2010 Mar 30;55(13):1310–7

(3) Echouffo-Tcheugui JB, Selvin E. Prediabetes and What It Means: The Epidemiological Evidence. Annual Review of Public Health. 2021;42(1):59–77.

14. **The authors should expand on the clinical relevance of their findings. How can clinicians evaluate and address vascular dysfunction in clinical practice to mitigate the risk of adverse cardiometabolic outcomes in patients with prediabetes?**

**Response**

We appreciate this point. Measuring vascular dysfunction in clinic is challenging for clinicians, both due to time constraints and logistical difficulties. However, we believe that our research has valuable implications for the design of future clinical trials. First, understanding that those with prediabetes exhibit a degree of vascular dysfunction emphasizes the importance of pursuing interventions that may reduce risk in individuals with prediabetes. Second, the strong association between measures of vascular dysfunction and MACE provides justification for the utilization of these measures as surrogate endpoints in pilot trials exploring potential interventions. If a potential intervention is found to improve BA-FMD or CAC, this would support larger more costly trials in individuals with prediabetes. We have clarified this in the discussion of the manuscript.

**Amendment**

Discussion paragraph 5:

“Interventions that target cardiovascular risk factors in adults with prediabetes, such as exercise, smoking cessation, and other lifestyle modifications in addition to pharmacologic therapies, may enhance vascular function in prediabetes allowing for early intervention (prior to the onset of overt diabetes and clinical CVD). The association of measures of vascular dysfunction with MACE in prediabetes suggest that the utilization of BA-FMD or CAC as surrogate outcomes may be appropriate to evaluate potential interventions in this patient population. Of note, interventions that improve vascular dysfunction have been shown to impact hard clinical outcomes such as CV events and/or mortality in other populations(41–46). A key strength of our study is the use of the Framingham Heart Study cohort, a well-characterized, longitudinal population study with standardized data collection, well-defined defined MACE, and robust vascular function assessments.”

15. **Finally, consider the potential impact of residual confounding on the results. Factors such as lifestyle, diet, and environmental exposures, which influence both vascular dysfunction and MACE events, should be discussed.**

**Response**

We agree that factors such as lifestyle, diet, and environmental exposures can influence both vascular function and MACE risk. Unfortunately, we are unable to account for these variables in our analysis. As such, we have noted this in the discussion as a limitation of the work.

**Amendment**

Discussion paragraph 6:

“Our study also did not evaluate the impact of lifestyle modifications such as exercise or diet, and environmental factors on the prediction of MACE using vascular function in prediabetes. These factors are known to influence vascular function and therefore may influence this relation”

**Reviewer 1**

1. **Abstract: Which CVD risk factors**

**Response**

Due to the abstract word limit, we were unable to include the full list cardiovascular risk factors in the results or methods section of our abstract. However, this is available both in the methods and results section of our manuscript. We hope this is acceptable to the reviewer.

2. **Introduction: What does a non-traditional risk factor mean? Versus traditional?**

**Response**

Traditional cardiovascular risk factors for cardiovascular disease include those that were established in the Framingham Heart Study, including blood pressure, cholesterol, smoking, age, sex, and obesity. Non-traditional risk factors for cardiovascular disease include inflammation, vascular function, lifestyle factors such as exercise, and other factors not captured in the original Framingham definition. We have amended the wording for clarity.

**Amendment**

Introduction Paragraph 1:

“However, even after adjusting for traditional cardiovascular risk factors, established in the Framingham Heart Study, residual CVD risk persists (5–7).”

3. **The observed association between prediabetes and MACE was attenuated after adjusting for traditional risk factors, which was attributed to sample size limitations. How could the limited sample size have affected the findings, did power calculations suggest insufficient statistical power?**

**Response**

We agree with the reviewer that sample size limitations are not a likely factor. Thus, we have deleted “possibly due to the limited sample size”.

4. **You mention that their sample was predominantly white and that this may limit generalizability. Could you comment on the potential impact of race and ethnicity on vascular dysfunction and MACE risk in prediabetes, perhaps referencing studies that have shown different cardiovascular risk profiles across racial groups?**

**Response**

There is conflicting evidence on vascular function differences by racial group. For instance, while Campia et al 2002 found reduced vascular function in African Americans, they did not consider environmental or lifestyle factors in their analysis, and other studies have shown no difference in vascular function between African Americans and whites (1,2). There is also no evidence we are aware that shows genetic differences in vascular function by race. However, there are known disparities in cardiovascular risk that continue to disproportionately affect minorities and people of color (47). There are often differing opportunities offered to people of different races, whether in neighborhood, access to exercise, and differences in medical care and preventative care (47). Such lifestyle factors are known to influence vascular function (16,41). This is what we sought to emphasize when we noted that our predominantly white cohort does not fully generalize to all populations. We may be over or underestimating the predictive capabilities of vascular function and future studies that capture participants with a wider range of backgrounds are needed to fully understand this interplay. We have expanded our discussion to reflect our meaning more accurately and we have incorporated additional references as suggested by the reviewer.

**Amendment**

Discussion paragraph 6:

“Since our study population does not reflect the full demographic diversity of the United States, our findings may not fully capture the CVD risk across different racial and ethnic groups. CVD risk is known to be higher in minority groups and both CVD risk and vascular function are impacted by access to social supports promoting exercise and other lifestyle modifications (16,41,47).”

References:

(1) Campia U, Choucair WK, Bryant MB, Waclawiw MA, Cardillo C, Panza JA. Reduced endothelium-dependent and -independent dilation of conductance arteries in African Americans. Journal of the American College of Cardiology. 2002 Aug 21;40(4):754–60.

(2) Perregaux D, Chaudhuri A, Rao S, Airen A, Wilson M, Sung BH, et al. Brachial Vascular Reactivity in Blacks. Hypertension. 2000 Nov;36(5):866–71

(16) Metsämarttila E, Rodilla E, Jokelainen J, Herrala S, Leppäluoto J, Keinänen-Kiukaanniemi S, et al. Effect of physical activity on pulse wave velocity in elderly subjects with normal glucose, prediabetes or Type 2 Diabetes. Sci Rep. 2018 May 23;8(1):8045.

(41) Qiu S, Cai X, Yin H, Sun Z, Zügel M, Steinacker JM, et al. Exercise training and endothelial function in patients with type 2 diabetes: a meta-analysis. Cardiovasc Diabetol. 2018 May 2;17(1):64.

(47) Javed Z, Haisum Maqsood M, Yahya T, Amin Z, Acquah I, Valero-Elizondo J, et al. Race, Racism, and Cardiovascular Health: Applying a Social Determinants of Health Framework to Racial/Ethnic Disparities in Cardiovascular Disease. Circulation: Cardiovascular Quality and Outcomes. 2022 Jan;15(1):e007917.

5. **Given that there is the limitation of not including menopause status in their study, which is important due to the known effects of menopause on vascular health. It would be helpful if you could suggest how future studies could account for hormonal factors or explore this further in prediabetic women. For example, a follow-up study could stratify by menopause status or investigate hormonal therapy as a potential modulator of vascular dysfunction.**

**Response**

We agree that it is important for follow up studies to evaluate the effect of pre and post-menopausal status on vascular function and MACE risk in prediabetes. We have provided guidance in our conclusion on ways that future researchers could evaluate this research gap.

**Amendment**

Conclusion Paragraph 6:

“Future studies could stratify participants by menopausal status to further evaluate the effects of vascular function on MACE risk in premenopausal and postmenopausal women.”

6. **I might suggest that the authors discuss how their results could influence existing treatment guidelines for prediabetes and CVD prevention. Would interventions targeting vascular dysfunction be integrated into current prediabetes management strategies, or is further evidence needed before such recommendations are made?**

**Response**

Thank you for raising this point. We do not believe that our findings justify targeting vascular function in clinical practice. However, the novel association between measures of vascular function and MACE in individuals with prediabetes is important for 2 reasons. First, it highlights the increased CVD risk in prediabetes and the importance of identifying interventions that might reduce this risk. Second, these data suggest that it may be appropriate to utilize measures of vascular function as surrogate outcomes in clinical trials. We have clarified this in the revised discussion.

**Amendment**

Discussion paragraph 5:

“Interventions that target cardiovascular risk factors in adults with prediabetes, such as exercise, smoking cessation, and other lifestyle modifications in addition to pharmacologic therapies, may enhance vascular function in prediabetes allowing for early intervention (prior to the onset of overt diabetes and clinical CVD). The association of measures of vascular dysfunction with MACE in prediabetes suggest that the utilization of BA-FMD or CAC as surrogate outcomes may be appropriate to evaluate potential interventions in this patient population. Of note, interventions that improve vascular dysfunction have been shown to impact hard clinical outcomes such as CV events and/or mortality in other populations(41–46). A key strength of our study is the use of the Framingham Heart Study cohort, a well-characterized, longitudinal population study with standardized data collection, well-defined defined MACE, and robust vascular function assessments.”

7. **The paper could also expand on the potential for early interventions. For example, could lifestyle interventions like exercise or dietary changes have a meaningful impact on vascular function in people with prediabetes before they progress to full diabetes?**

**Response**

The reviewer raises an interesting and important point. As noted in the amendment above, we have incorporated this point into the discussion.

**Reviewer 2**

Thank you for the your effort and contribution to the field with this study. Your work on a topic of such clinical importance is appreciated. Below are some suggestions to enhance the clarity and rigor of the manuscript:

1. **Although the authors mention the Framingham cohorts later in the text, they fail to justify why this dataset is particularly well-suited to address their research question. The manuscript would benefit greatly from a compelling rationale for selecting these cohorts. Why should the reader consider the use of this cohort as a significant strength of the study>**

**Response**

The Framingham Heart Study is a well characterized study in which MACE is well defined and vascular function measurements are robust. We have added this to the conclusion.

**Amendment**

Conclusion paragraph 6:

“A key strength of our study is the use of the Framingham Heart Study cohort, a well-characterized, longitudinal population study with standardized data collection, well-defined defined MACE, and robust vascular function assessments.”

2. **The authors state that data are available through BioLINCC but fail to provide a direct link or accession details. This lack of transparency makes it challenging for other researchers to verify or reproduce the results.**

**Response**

Unfortunately, we are unable to provide a direct link for access. The Framingham Heart Study and BioLINCC both require IRB approval and data use agreements prior to the release of even the de-identified data. Researchers who would like access to the data set will need to obtain IRB approval in order to obtain access to the data set. Clarification was added to the materials and methods section.

Materials and methods study design:

“This study was approved by the University of Iowa Human Subjects Office and Institutional Review Board (IRB 201811756). As de-identified data was obtained from BIOLINCC, a waiver of informed consent was provided.”

3. **The Results section does not address potential interaction effects between key variables, such as age, sex, or prediabetes criteria and vascular dysfunction. This is a significant oversight in a study aiming to examine independent associations. Were vascular dysfunction measures equally predictive of MACE across subgroups?g**

**Response**

Thank you for raising this point. As such, we have added interaction terms to evaluate the relation between key variables including age, sex, or prediabetes definition (HbA1c or fasting glucose criteria met) and vascular dysfunction as suggested. The details of this analysis are explored above in response to editor comments. In summary, the inclusion of these interaction terms did not alter the relation between measures of vascular dysfunction and MACE. BA-FMD and CAC >100 continued to significantly predict the MACE in prediabetes.

4. **The dramatic reduction in the hazard ratios, such as for CAC > 100 (from 11.3 unadjusted to 3.90 after adjustment) is not adequately explained. This substantial attenuation suggests major confounding factors, yet the authors fail to critically examine or discuss this.**

**Response**

We agree with the reviewer that this attenuation is likely due to adjustment for confounding variables, such as those that we included in our model. The variables adjusted for included age, sex, smoking status, systolic blood pressure, BMI, and LDL. Additionally, per editor recommendation, we have added eGFR to the model. After this adjustment, the adjusted hazard ratio and 95% confidence interval were 4.15 (2.24, 7.70), p<0.001. While there is reduction of the HR in comparison to the unadjusted model, significance remains. As noted in comments above, we also evaluated the relation between key variables including age, sex, or prediabetes definition (HbA1c or fasting glucose criteria met) and vascular dysfunction using interaction terms; the inclusion of these interaction terms did not alter the significance of the relation between CAC score and MACE in prediabetes.

**Amendment**

See Table 3.

Discussion Paragraph 1:

“Additionally, endothelial dysfunction and CAC score > 100 were associated with future MACE in prediabetes after adjusting for confounding variables.”

5. **The authors imply causation between vascular dysfunction and MACE in prediabetes, despite their study being observational in design. For example, they state: “Our findings suggest that the elevated CVD risk observed in prediabetes may be mediated, in part, by vascular dysfunction” Such claims must be tempered, with explicit acknowledgment of the limitations of causal inference in the context of Cox regression analysis.**

**Response**

We agree that as an observational study we cannot make casual inferences from our data. With this in mind, we have tempered such claims throughout the paper.

**Amendment**

Paragraph 1 of conclusion:

“These data indicate that these measures of vascular dysfunction predict MACE in individuals with prediabetes.”

6. **While the authors suggest that therapies targeting vascular dysfunction may reduce MACE in prediabetes, they do not provide specific recommendations or actionable insights. How do their findings translate into clinical practice? What are the next steps for researchers and clinicians? Providing clarity here would enhance the utility of the study.**

**Response**

Thank you for raising this point. As noted above in response to Reviewer 1, we do not believe that our findings justify targeting vascular function in clinical practice. However, our findings highlight the increased CVD risk in prediabetes, the importance of identifying interventions that might reduce this risk and suggest that it may be appropriate to utilize measures of vascular function as surrogate outcomes in clinical trials. We have incorporated this into the revised discussion.

**Amendment**

Discussion paragraph 5:

“Interventions that target cardiovascular risk factors in adults with prediabetes, such as exercise, smoking cessation, and other lifestyle modifications in addition to pharmacologic therapies, may enhance vascular function in prediabetes allowing for early intervention (prior to the onset of overt diabetes and clinical CVD). The association of measures of vascular dysfunction with MACE in prediabetes suggest that the utilization of BA-FMD or CAC as surrogate outcomes may be appropriate to evaluate potential interventions in this patient population. Of note, interventions that improve vascular dysfunction have been shown to impact hard clinical outcomes such as CV events and/or mortality in other populations(41–46). A key strength of our study is the use of the Framingham Heart Study cohort, a well-characterized, longitudinal population study with standardized data collection, well-defined defined MACE, and robust vascular function assessments.”

7. **Providing a legend for the figure would help readers interpret the figures more effectively.**

**Response**

The following legend is provided in the manuscript after the first reference of the figure:

“Fig 1: Vascular function and CAC score in Framingham.

A. BA-FMD in normoglycemic participants and participants with prediabetes in the Framingham cohort. Data reported as median and interquartile range. p≤0.05 B. cfPWV in normoglycemic participants and participants with prediabetes in the Framingham cohort. Data reported as median and interquartile range. p≤0.05 C. CAC score in normoglycemic participants and participants with prediabetes in the Framingham cohort. Data is reported as the percentage of participants per group. p≤0.05 All figure p values reported refer to differences in normoglycemia and prediabetes groups.”

**Reviewer 3**

Research has proven that prediabetes is a risk for CVD. Vascular dysfunction is a non-traditional risk factor that increases CVD risk. Measures of vascular function such as BA-FMD, cfPWV, and CAC score have been associated with an elevated risk of cardiovascular events and all-cause mortality. The authors, therefore, conducted an observational study to assess the relationship between vascular dysfunction and the risk of MACE in prediabetes. The study design and tests align with the study's objectives. The results were presented logically and are sufficient to conclude that vascular dysfunction is independently associated with MACE in prediabetes.

Results section
**I suggest that the authors include subheadings for the results so the reader can easily follow and understand the order of the results e.g, a subheading before line 178 where the authors present results on BA-FMD, cfPWV and CAC score. A subheading before line 197**

**Response**

We have added subheadings to the results section to enhance the clarity of the manuscript, as per reviewer suggestion.

**Amendment**

The following subheading titles were added: “Participant characteristics”, “Vascular function and CAC score in prediabetes”, Variables associated with vascular function in prediabetes”, and “MACE in prediabetes”.
